# Management of Therapeutic-intensity Unfractionated Heparin: A Narrative Review on Critical Points

**DOI:** 10.1055/a-2359-0987

**Published:** 2024-10-17

**Authors:** Isabelle Gouin-Thibault, Alexandre Mansour, Michael Hardy, Pierre Guéret, Emmanuel de Maistre, Virginie Siguret, Adam Cuker, François Mullier, Thomas Lecompte

**Affiliations:** 1 Department of Laboratory Hematology, Pontchaillou University Hospital of Rennes, France; 2 IRSET-INSERM-1085, Univ Rennes, Rennes, France; 3 Department of Anesthesia and Critical Care, Pontchaillou University Hospital of Rennes, France; 4 Department of Biology, Université Catholique de Louvain, CHU UCL Namur, Namur Thrombosis and Hemostasis Center, Namur Research Institute for Life Sciences, Hematology Laboratory, Yvoir, Belgium; 5 Department of Anesthesiology, Université catholique de Louvain, CHU UCL Namur, Namur Thrombosis and Hemostasis Center, Namur Research Institute for Life Sciences, Yvoir, Belgium; 6 Division of Laboratory Hematology, University Hospital of Dijon Bourgogne, Dijon, France; 7 AP-HP, Department of Laboratory Hematology, University Hospital of Lariboisière, INSERM UMRS-1140, Paris Cité University, Paris, France; 8 Department of Medicine and Department of Pathology and Laboratory Medicine, Perelman School of Medicine, University of Pennsylvania, Philadelphia, Pennsylvania, United States; 9 Department of Biology, Université Catholique de Louvain, Institut de Recherche Expérimentale et Clinique—Pôle Mont, Namur, Belgium; 10 Department of Pharmacy, Namur Thrombosis and Hemostasis Center, Namur Research Institute for Life Sciences, University of Namur, Namur, Belgium; 11 Division of Vascular Medicine, University Hospital of Nancy, University of Lorraine, Nancy, France

**Keywords:** heparin, aPTT, anti-Xa, antithrombin, nomogram, drug resistance

## Abstract

Nowadays, unfractionated heparin (UFH) use is limited to selected patient groups at high risk of both bleeding and thrombosis (patients in cardiac surgery, in intensive care unit, and patients with severe renal impairment), rendering its management extremely challenging, with many unresolved questions despite decades of use.

In this narrative review, we revisit the fundamental concepts of therapeutic anticoagulation with UFH and address five key points, summarizing controversies underlying the use of UFH and discussing the few recent advances in the field: (1) laboratory tests for UFH monitoring have significant limitations; (2) therapeutic ranges are not well grounded; (3) the actual influence of antithrombin levels on UFH's anticoagulant activity is not well established; (4) the concept of UFH resistance lacks supporting data; (5) scarce data are available on UFH use beyond acute venous thromboembolism.

We therefore identified key issues to be appropriately addressed in future clinical research: (1) while anti-Xa assays are often considered as the preferred option, we call for a vigorous action to improve understanding of the differences between types of anti-Xa assays and to solve the issue of the usefulness of added dextran; (2) therapeutic ranges for UFH, which were defined decades ago using reagents no longer available, have not been properly validated and need to be confirmed or reestablished; (3) UFH dose adjustment nomograms require full validation.

## Introduction

Unfractionated heparin (UFH) has been used in humans since the 1930s. Therapeutic doses are required to treat acute thromboembolic events to prevent an increase in the thrombotic burden and to facilitate endogenous thrombolysis. 1 2 It was considered that such doses should induce a hypocoagulable state detectable by a laboratory clotting time, i.e., the activated partial thromboplastin time (aPTT)—the so-called anticoagulation effect. 3

Nowadays, with the advent of low molecular weight heparin (LMWH) and rapidly acting oral anticoagulants, UFH use for the management of acute venous or arterial thrombosis, or for the prevention of thrombotic events in settings with high risk of events, is limited to selected patient groups: patients in cardiac surgery, in intensive care unit, and patients with severe renal impairment. Patients exposed to those conditions often suffer from inflammation and have a hypercoagulable state, characterized by the presence of circulating coagulation activators and/or a hyperresponsive coagulation system, leading to excessive thrombin generation (TG) and/or increased fibrin mass. 4 5 Since they are often at high risk of bleeding as well, their management is highly challenging.

Heparin, a linear glycosaminoglycan defined by the nature of its basic disaccharide unit, is a heterogeneous mixture of negatively charged (sulfated) chains of different lengths.

The beneficial clinical effects of UFH are foremost ascribed to anticoagulation, mediated by chains that interact with high affinity with antithrombin (AT), thanks to a peculiar pentasaccharide present only in about one-third of chains. The inhibitory effect of AT on activated serine-proteinases, such as IIa, Xa, and others, is accelerated by heparin. 2 A minimal chain length (≥18 saccharides units including the pentasaccharide sequence; ≥5,400 Da) is required for acceleration of the inhibition of thrombin (IIa) by AT. 2 6 7 The respective intensities and roles of the inhibition of the different serine-proteinases are still debated; thrombin being the most sensitive to inhibition by approximately an order of magnitude. 7 While the labeling of UFH in International Units (IU) suggests standardization, UFH preparations may differ in terms of bulk material and proportion of chain lengths. The half-life of UFH is dose-dependent, ranging from 60 to 90 minutes at usual intravenous (IV) doses. 8

The lengths of the chains determine their ability to interact with many proteins, the so-called interactome; such interactions divert heparin from interacting with AT and are the molecular basis of its pleiotropic effects. 4 5 9 10 Interactions with many proteins and cells of the human body result in complicated UFH pharmacokinetics (PK) and pharmacodynamics and explain the large inter- and intraindividual variability in response to UFH. 7 Hence, laboratory monitoring of therapeutic UFH is advocated with repeated laboratory testing to assess the anticoagulant response to UFH and adjust the dose, i.e., laboratory monitoring. 8

For the purposes of this manuscript, the term “therapeutic dose” refers to doses used for treatment of acute venous thromboembolism (VTE), with the accompanying term “therapeutic range.” Therapeutic-intensity UFH is also used to prevent clotting on the foreign surface of mechanical heart valves, circulatory assist devices (extracorporeal membrane oxygenation [ECMO]), or extracorporeal circuits. 11 12 We define the “anticoagulant effect” as either the prolongation of a clotting time (aPTT) or the inhibitory effect on an activated coagulation serine-proteinase, such as factors IIa or Xa. With the term laboratory tests, we refer to aPTT and anti-Xa assay, unless otherwise specified.

**Fig. 1:**
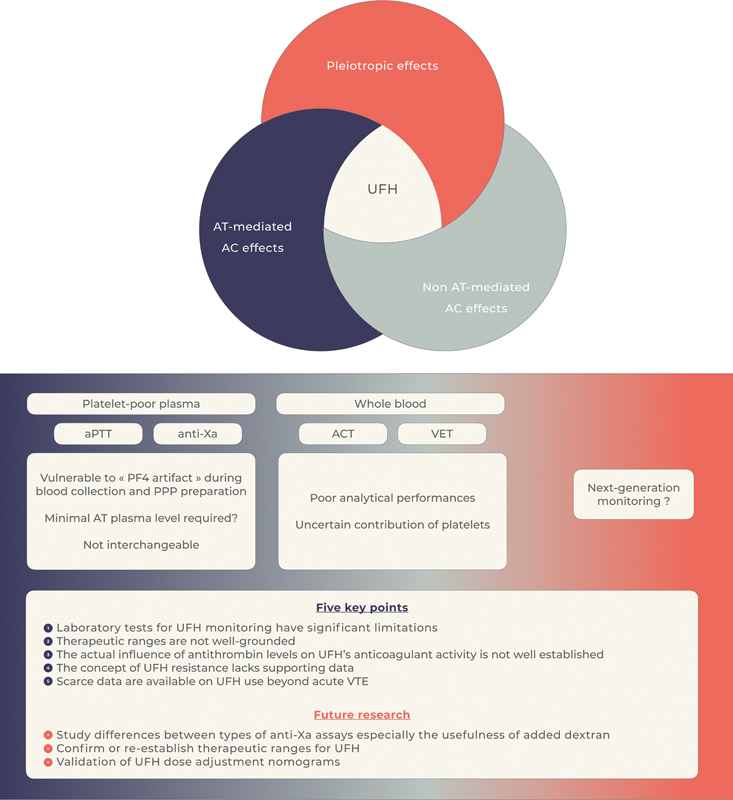
Pleiotropic effects of UFH and laboratory tests for its monitoring in platelet-poor plasma and whole blood. Abbreviations: ACT, activated clotting time; aPTT, activated partial thromboplastin time; AT, antithrombin; AC, anticoagulant; PF4, platelet factor 4; UFH, unfractionated heparin; VET, viscoelastometric tests.

In this narrative review, we revisit crucial fundamental concepts, which must be fully considered when thinking over the still unresolved questions regarding UFH therapy and especially laboratory monitoring ( Fig. 1 ). We expand upon and complement some issues previously addressed by others. 2 6 13 14 15 16 We propose key issues to be appropriately addressed in future clinical research.

## Laboratory Tests for Unfractionated Heparin Monitoring have Significant Limitations (Key Point #1)

First, the pleiotropic effects of UFH, which may have clinical relevance, are not investigated with aPTT and anti-Xa. The effects of UFH heavily rely on, but are not restricted to, anticoagulation mediated by AT. The antithrombotic properties of UFH could also be supported by several AT-independent mechanisms, such as the catalysis of thrombin inhibition by heparin cofactor II (HCII) and the mobilization of endogenous tissue factor pathway inhibitor from the endothelium. 7 17 Non AT-mediated effects may impact the hemostatic system and beyond, being either beneficial (“anti-inflammatory,” “antiproliferative,” “antiadhesive effects”…) or harmful. 4 10 17 18 However, the exact molecular mechanisms that mediate those effects of heparin remain poorly understood. 10 More specifically, regarding the interactions of UFH with platelets, a “proaggregatory” effect has been widely proposed, but impairment of collagen-induced aggregation and of adhesion to collagen has also been reported. 19 20 In addition, since heparins have an inhibitory effect on thrombin, they inhibit thrombin-induced platelet activation. 21

Second, coagulation tests are performed with platelet-poor plasma and hence miss the role of platelets in coagulation. Platelets contain platelet factor 4 (PF4), a heparin-inhibiting protein, in their α-granules. PF4 can be released into plasma in the collected blood before testing and therefore reduce the anticoagulant effect of UFH, which is a cause of underestimation. One method to prevent ex vivo platelet activation and release of heparin neutralization proteins is to collect blood into tubes containing a mixture of citrate and the platelet inhibitory cocktail of theophylline–adenosine–dipyridamole (CTAD solution) instead of citrate alone. 22 23 24 However, the extent to which this mixture limits platelet activation, PF4 release, and heparin neutralization, remains unclear. In some studies, only small differences in anti-Xa levels, between samples collected in CTAD versus citrate tubes, 24 25 26 were reported, whereas in the DEXHEP study, higher levels from +8 and up to +24% were found, depending on the clinical setting. 27 Thus, for some blood samples, PF4 release in the collecting tube could be a problem. Double centrifugation minimizes PF4 availability after thawing frozen plasma samples, since it results in a smaller number of residual platelets but may increase PF4 release during the process of plasma preparation, especially when high speed is used. 28 29

During the course of coagulation, the first tiny amounts of thrombin potently activate platelets, before any change in the physical properties of plasma is detected. Once activated, platelets support coagulation by providing a suitable surface enriched in negatively charged phosphatidylserine, for the assembly of the intrinsic tenase and prothrombinase complexes, responsible for the thrombin burst. Coagulation tests are performed with procoagulant phospholipid vesicles of various natural origins, which form the “partial thromboplastin” portion of aPTT reagents. Their nature, which is often proprietary and therefore undisclosed, is complex and differs between reagents. They are a poor substitute for activated platelets and their role in coagulation, which extends well beyond procoagulant phospholipids and PF4 release (e.g., factor V released from α-granules, binding and protecting factor Xa from inactivation by UFH–AT). 7 30 The exogenous phospholipids do not even faithfully mimic platelet phospholipid surfaces, which change over time during the coagulation process. In short, contribution of platelets to TG relies on phosphatidylserine exposure but is not restricted to this phenomenon. The anticoagulant effects of UFH are therefore substantially reduced when studied in the presence of platelets, as compared with platelet-poor plasma with nonplatelet procoagulant phospholipid surfaces. 31 The issue could be even more complicated taking into account active tissue factors made available on the surface of activated platelets, according to some reports. 30

Platelets are present in whole blood tests like viscoelastometric assays 32 and activated clotting time (ACT), but this does not imply that their entire physiological role in coagulation is faithfully represented in those tests. 33 Such tests are widely used in the management of patients in the context of cardiac surgery with cardiopulmonary bypass or other procedures, when blood comes into contact with foreign surfaces with large doses of UFH 11 (ACT), but also in postprocedure for viscoelastometric assays. In contrast, many limitations prevent their use to monitor therapeutic UFH and their reliability in this context remains highly questionable. 34

So far, in the absence of a validated genuinely integrative (“global”) approach, UFH monitoring must rely on the use of the plasma-based tests, aPTT clotting times, or anti-Xa assays. Both tests may be performed within 1 hour, with organized efforts to expedite the central laboratory turnaround time similar to those done in emergency settings. 35 Despite the lack of robust validation, anti-Xa levels have often been considered a better option for UFH monitoring than resorting to aPTT. 13 36 37

### Limitations Specific to Activated Partial Thromboplastin Time Tests

By contrast with anti-Xa assays, aPTT explores the coagulation cascade initiated with contact phase. It is highly sensitive to many conditions unrelated to the anticoagulant effect of UFH, frequently encountered in critically ill patients. These include defects in the contact system, interference with C-reactive protein with certain aPTT reagents, and presence of a lupus anticoagulant, which prolong aPTT. Conversely, high factor VIII levels, which are frequent in case of inflammation, shorten the aPTT thus rendering it less sensitive to UFH. 8 14 36 38 39 Moreover, the aPTT is not or only poorly sensitive to natural anticoagulants (see below regarding AT, key point #3).

Due to the variability of reagent–coagulometer sensitivity to UFH, 8 36 40 41 the American College of Chest Physicians (ACCP) consensus group recommended against the use of a fixed aPTT therapeutic range in seconds and suggested that the therapeutic range be calibrated, specifically for each reagent batch/coagulometer, against anti-Xa levels (0.30–0.70 IU/mL), 8 42 which is a difficult task, seldom performed. This ex vivo calibration may be impacted by the choice of a specific anti-Xa assay (see below) 43 and has not been found to enhance interlaboratory agreement in UFH monitoring. 13 44 Also, it has never been demonstrated to be associated with better clinical outcomes, as compared with the traditional 1.5 to 2.5 times control method. 13 44 Moreover, it is unclear whether the control value should be: (1) the one of a pool of normal plasma samples (how should this be prepared? Are commercially available samples suitable?), (2) the geometric mean of values of a sufficient (how many?) of normal plasma samples, or (3) baseline value of the patient. 3 Anyhow, the patient baseline value is required to identify patients with prolonged aPTT due to disorders not associated with bleeding, such as lupus anticoagulant and contact phase abnormalities. In those occurrences, one has to resort to an anti-Xa assay.

Despite these limitations, the use of aPTT instead of the anti-Xa assay for UFH monitoring continues to be widespread due to cost and availability issues. 2 6 Indeed, the per-test cost anti-Xa assay, which varies among countries, is higher than that of aPTT. 45 46 47

### Limitations Specific to Anti-Xa Assays

In contrast to aPTT, anti-Xa assays may be considered as fairly standardized. However, limited agreement between anti-Xa assays has been repeatedly reported, with potential important clinical impact leading to changes in treatment decisions. 37 43 48 49 For instance, in patients receiving therapeutic UFH, discrepancies in anti-Xa levels within the therapeutic range (0.30–0.70 IU/mL) reached up to 46% when comparing two reagents, BiophenLRT (Hyphen) and STA-Liquid anti-Xa (Stago). 48 One substantial difference among anti-Xa assays is the presence or absence of dextran sulfate (DS) in the assay. The type of DS (molecular weight, degree of sulfation…) and its concentration can vary among manufacturers and is usually undisclosed. DS is used to partly displace UFH from proteins released in vitro into plasma after blood sampling by platelets, especially PF4, to recover UFH activity. 50 51 DS also likely displaces UFH from complexes formed in vivo with various proteins (the so-called interactome), or with protamine after UFH neutralization in the setting of cardiac surgery under cardiopulmonary bypass, contributing to higher anti-Xa levels and probably to overestimation of the actual anticoagulant activity of UFH. 27 37 48 49 51 52 For instance, after protamine neutralization of UFH in cardiac surgery, only 6% of anti-Xa values versus 77% were found below the lower limit of quantification as expected, when measured with reagents containing DS or without DS, respectively. 27 Potentially clinically relevant discrepancies between anti-Xa values, measured with or without DS, were also observed in patients receiving ECMO therapy. 53

In addition to the presence of DS, other parameters might potentially contribute to disparities in anti-Xa levels, including the type of blood collection tube, the addition of exogenous AT, the calibrator, and the calibration curve mathematical processing. 37 Much effort must be made to improve the understanding of the differences between types of anti-Xa assays, 27 51 which requires a concerted action by professional societies and reagent manufacturers. To the best of our knowledge, the relationship with Stachrom Heparin-derived values, used to established the 0.30 to 0.70 IU/mL therapeutic range as mentioned below, 54 55 is not available for any of the currently used anti-Xa assays. There are theoretical reasons, mentioned above, to opt for an assay without DS (Stago, Technoclone, Horiba). However, extended studies about the clinical impact of their use and even their analytical performances are lacking.

Moreover, anti-Xa levels provide no information about the effect of UFH in case of inflammation and hypercoagulability (with elevated factor VIII among other changes), which is a cause of dissociation between the two assays, with anti-Xa level in the therapeutic range but aPTT below the therapeutic range. 56

In addition, coagulopathy due to liver impairment, trauma, postsurgical bleeding, and disseminated intravascular coagulation and coagulation factor deficiencies of any cause, should not be overlooked; they can be detected with PT and fibrinogen determination.

One may wonder why UFH levels are estimated using the potential of UFH in plasma to inhibit added Xa rather than IIa, which is likely the main target in the anticoagulant effect of UFH. 31 Actually, the anti-Xa effect is assessed in a very artificial manner. In those assays, Xa is entirely in the fluid phase and not in the prothrombinase complex, which partially protects Xa from AT inhibition. Moreover, calcium concentrations are low, artificially increasing the effect of heparins against factor Xa. 57 Nevertheless, anti-Xa levels are considered a good surrogate for anti-IIa levels in UFH management. By definition, the anti-Xa/anti-IIa ratio (in “units”) is one for UFH, as almost if not all the chains in UFH that contain the pentasaccharide with high affinity for AT are long enough to inhibit factor IIa. This does not mean that they are equally active against (free) IIa and (prothombinase) Xa. Thus, anti-Xa levels should closely match anti-IIa levels, as far as the inhibitory effect is mediated by AT. The anti-IIa assay is not used in daily practice for various practical reasons and also because anti-Xa assays can capture other widely used anticoagulants (LMWH, fondaparinux, danaparoid, and direct oral Xa inhibitors), which is a potential source of confusion when a patient is switched from a direct oral Xa inhibitor to UFH.

By design, anti-Xa assays do not assess the effect of UFH on HCII, 58 the in vivo role of which is not well defined. 59

Finally, to what extent the different formats of the anti-Xa assay are sensitive to endogenous circulating anticoagulant glycosaminoglycans is not clear. 60 61

To summarize, the respective advantages/disadvantages of aPTT and anti-Xa assays are displayed in Table 1 . So far, there are too few data to conclude on the clinical implications of aPTT or anti-Xa assay-based UFH monitoring (see below). However, it has been reported that the use of an anti-Xa assay, rather than aPTT, is associated with a faster time to reach the therapeutic range (see below). 47 54 62 63

**Table 1:** Respective advantages/disadvantages of activated partial thromboplastin time and anti-Xa

	aPTT	Anti-Xa
Availability	Widely available	Not widely available
Cost	Cheap	Expensive
Principle	Clotting time after activation of the contact system	Target-specific test
Analytical interferences	Prolongation in case of:	
	• Lupus anticoagulant • Interference from CRP • Deficiency in coagulation factors (contact system)	• Interference from other anti-Xa anticoagulants
	Shortening in case of inflammation	
Sensitivity to UFH	Highly variable, depends on reagents	Sensitive But lack of standardization of different assays can lead to large deviations in the levels
Specificity	No	Specific to anti-Xa inhibitors

Abbreviations: aPTT, activated partial thromboplastin time; CRP, C-reactive protein; UFH, unfractionated heparin.

## Therapeutic Ranges are not Well Grounded (Key Point #2)

The anti-Xa range from 0.3 to 0.7 IU/mL has been widely accepted for decades as the UFH therapeutic range for the treatment of acute VTE. 8 However, the method by which the range was established should be viewed with skepticism, as should the strength of the relationship between the intensity of anticoagulation and clinical outcomes (thrombosis and bleeding).

The substantial inter- and intraindividual variability of the response to UFH administration was appreciated decades ago. 64 This led to the use of laboratory monitoring for dose adjustment with the tools available at that time, many years before anti-Xa chromogenic assays became available. 14 The UFH therapeutic range for an acute episode of VTE was first established as an aPTT ratio between 1.5 and 2.5 times control (pooled normal plasma, 40 seconds). 3 More specifically, the prospective study performed in the 1970s with 234 patients (162 with VTE and 72 with myocardial infarction or arterial thrombosis) treated with an initial bolus followed by a continuous IV infusion, suggested that an aPTT ratio between 1.5 and 2.5 was associated with a low risk of recurrent thrombosis. 3 aPTT was measured in citrated platelet-poor plasma with the use of kaolin (contact phase activator) and a 3-minute incubation time before recalcification together with the addition of phospholipids (“partial thromboplastin”). Such aPTT methods with locally prepared reagents are not used anymore.

Thereafter, heparin levels were also assessed with protamine titration, relying on experimental animal studies 65 and subsequently patient samples, after IV administration for VTE treatment, but without assessment of clinical outcomes. 66 In one study, Brill-Edwards et al showed that establishing a therapeutic range, using protamine titration heparin levels of 0.2 to 0.4 units/mL as a reference was feasible and compensated for the variable response of aPTT reagents to heparin. 66 The transition from protamine titration to chromogenic anti-Xa assay was based on a single study performed by the same Canadian group, on VTE patients requiring large daily doses of UFH (≥35,000 IU) with assessment of recurrence and bleeding events, which enabled the establishment of a heparin level therapeutic range of 0.35 to 0.67 IU/mL. 54

Patients requiring a large daily dose of UFH were randomized to aPTT or anti-Xa monitoring, with therapeutic ranges corresponding to 0.2 to 0.4 units/mL protamine titration, i.e., 60 to 85 seconds for aPTT (Actin FS, Dade) and 0.35 to 0.67 IU/mL for anti-Xa (Stachrom Heparin, Stago). The mean daily heparin doses were higher in the aPTT group compared with the anti-Xa group. During the first 12 weeks, 3/65 and 4/66 patients experienced recurrent VTE in the anti-Xa and aPTT groups, respectively (difference: 1.5%; 95% confidence interval [CI]: −6.7 to 8.4%; *p* = 0.7). There were four bleeding events in the aPTT group and one in the anti-Xa group (difference, 4.6%; 95% CI: −3.3 to 7.5%; *p* = 0.4).

The anti-Xa reagent used at that time, Stachrom Heparin (Stago), a dextran–free reagent with added AT, is seldom used nowadays, and the applicability of the results to current widely used anti-Xa assays is unknown. 55 Since the seminal studies, no clinical studies have challenged the therapeutic range (rounded to 0.3–0.7 IU/mL) and no evidence has been produced, in a large cohort of patients, demonstrating that certain UFH levels, as reflected by current anti-Xa assays, are associated with optimal clinical outcomes. 13 16 Interestingly, while the aPTT range as defined in the Basu et al study 3 was not validated in clinical studies, the aPTT was nevertheless widely used, albeit with a great diversity of therapeutic ranges, in the UFH arms of pivotal studies aimed at comparing LMWH to UFH in the treatment of acute VTE. 14

At least two randomized trials suggest that the efficacy (early recurrence, extension of VTE) of UFH in the treatment of VTE is dependent on initial dosing and the levels of anticoagulation achieved within 24 to 48 hours. 13 67 68 Yet, the suboptimal result with intermittent subcutaneous heparin, compared with continuous IV UFH to reach the aPTT-target therapeutic range and to prevent recurrent VTE, in the initial treatment of patients with acute proximal deep vein thrombosis, was established in a randomized double-blind trial, with recurrences limited to patients with an initial subtherapeutic anticoagulant response. 69 In this study, the subcutaneous UFH regimen, deemed inadequate by contemporary standards, induced an initial anticoagulant response below the target therapeutic range in the majority of patients and resulted in a high frequency of recurrent VTE (11/57, 19.3%). In contrast, IV UFH induced a therapeutic anticoagulant response in the majority of patients and was associated with a low frequency of recurrent events (3/58; 5.2%; *p* = 0.024). 69

When considering subcutaneous UFH regimen, a fixed dose of 250 IU/kg every 12 hours, preceded by a 333 IU/kg SC loading dose, without monitoring, was shown to be noninferior to twice-daily LMWH with respect to recurrent VTE at 90 days (3.8 vs. 3.4%) and major bleeding (1.1 vs. 1.4%) in the randomized Fixed-Dose Heparin trial. 70 Whether this regimen, fixed-dose without monitoring has been used since this study, is unclear.

Few studies report clinical outcomes on the basis of UFH monitoring, using aPTT or anti-Xa. As above mentioned, in 1995, Levine et al were the first to show that anti-Xa monitoring could be used to monitor UFH. The authors concluded that the heparin assay is a safe and effective method, for monitoring heparin treatment in patients with acute VTE whose aPTT remains subtherapeutic despite large daily doses of heparin, avoiding dosage escalation if the anti-Xa level is therapeutic. 54

To our knowledge, only one review and meta-analysis aimed to evaluate if the choice of laboratory test for monitoring UFH therapy was associated with a difference in the outcomes of bleeding, thrombosis, or mortality, is available. 71 This review did not show any advantage when comparing aPTT-guided and anti-Xa-guided UFH therapy, with respect to bleeding or thrombotic events. However, there are important limitations in the review, related to the type of studies included, the criteria or definitions of bleeding across studies, and the therapeutic ranges. 71 Indeed, the number of patients in all but one study was small and the incidence of recurrence and bleeding was low. There was no consensus for the criteria or definitions of bleeding across studies. The aPTT reagents and anti-Xa assays used varied among studies as well as aPTT target ranges, whereas the anti-Xa target ranges were between 0.30 and 0.70 IU/mL in most studies. Importantly, one study represented the majority of the data analyzing bleeding and thrombotic complications. 71

## The Actual Influence of Antithrombin Levels on Unfractionated Heparin's Anticoagulant Activity is Not Well Established (Key Point #3)

AT deficiency can occur due to several causes, inherited or acquired, in the latter case due to reduced synthesis related to liver dysfunction or accelerated clearance/consumption associated with mechanical devices (cardiopulmonary bypass, ventricular assist device, ECMO), nephropathy (renal losses), disseminated intravascular coagulation, extended deep venous thrombosis/pulmonary embolism, or L-asparaginase administration. 11 72 Patients who are treated with UFH are likely exposed to those conditions. The question is whether AT deficiencies can impact the capacity of UFH to exert its anticoagulant activity in vivo and ultimately patient outcomes. It is important to note that anti-Xa assays supplemented with AT are, by design, insensitive to variations in endogenous AT levels.

It has been suggested that a AT level of 50 IU/dL (50%) should be sufficient to support a heparin effect assessed with anti-Xa assays that do not contain exogenous AT; however, no hard data support this assumption. 49 The very few in vitro studies available showed that the impact of AT would vary according to the assay used to monitor UFH, with no threshold to define a “resistance”/“altered response” to UFH. 73 74 Therefore, plasma level of AT required for the full desired anticoagulant effect of UFH is not known or established and likely depends on the patient's condition.

From a clinical point of view, the benefit of AT compensation for anticoagulation and for clinical outcomes, in case of altered laboratory response to UFH has not been documented, so far. Most available data concerning AT administration are from studies in cardiac surgery, only showing that AT supplementation may improve heparin reactivity measured by ACT. 11 In this context, randomized controlled trials failed to demonstrate any benefit of AT supplementation on patient outcomes, with rising concerns about a higher bleeding rate or incidence of acute kidney injury. 75

However, this does not mean that AT levels have no effect on the anticoagulant effect of UFH. This effect can be approached more comprehensively in a TG assay. It is important to understand that any AT levels can affect the thrombin potential in a TG assay and the decrease in thrombin potential afforded with UFH; the effect is continuous with no discernible threshold. 31 76 We thus do not dismiss that functional AT levels could impact the clinical outcome for a patient while on therapeutic UFH. Still, this impact is poorly or not detected at all by aPTT 74 or anti-Xa assays and clinical evidence is lacking.

## The Concept of Unfractionated Heparin Resistance Lacks Supporting Data (Key Point #4)

It is of utmost importance to differentiate the UFH response according to laboratory tests (the anticoagulant effect) from clinical outcomes: clinical failures do not entirely match failures to achieve the desired effect on laboratory tests. Here, we specifically address the laboratory side and challenge the widely held belief that “resistance” to UFH (frequently mentioned in the setting of coronavirus disease 2019 for instance 77 ) exists and warrants specific action.

Stricto sensu, resistance would mean that heparin could not act at all on its target in coagulation, AT, due to a cause that cannot be overcome. 78 Theoretically, severe AT deficiency or homozygous type II heparin-binding site inherited AT deficiency, prothrombin Belgrade 79 80 could lead to such a condition. 78 In case of acquired AT deficiency, such a resistance does not exist, whereas an attenuation of anticoagulation is very plausible at low AT levels, which can be readily overcome by increasing the UFH dose with the help of a nomogram.

If genuine resistance to UFH does not exist, as we believe (or is exceptional), it does not come as a surprise that there is no consensus on how to define it. For some patients, there is a need for high heparin doses, with various reported definitions, to achieve a targeted level of anticoagulation. 15 This is due to the large inter- and intraindividual variability in response to UFH, related to the interactome (see above) including increased release of PF4 in some acute phase setting, in association with the potential acquired AT deficiency, rather than to a genuine resistance. Therefore, despite the widespread use of the term “resistance,” we do not advocate for its use. “Resistance“ would imply that the ultimate target of heparin (i.e., thrombin) would not be affected at all by reasonable amounts of infused UFH, even after appropriate dose adjustments within the first 24 hours.

From a practical point of view, it is clinically important to promptly reach therapeutic levels of UFH within 24 hours, especially in case of acute VTE. 69 Appropriate monitoring with swift dose adjustments according to a reliable weight-adjusted nomogram should often solve the issue. 47 63 68 81 82 83 84 85 This is especially true if therapy is guided with an anti-Xa assay. 47 62 63

A randomized controlled trial conducted by Raschke et al in 1993 comparing weight-based nomogram and standard care groups showed that a significantly higher proportion of patients reached the aPTT therapeutic range of 1.5 to 2.3 times control within 24 hours, in the weight-based nomogram group, and that recurrent thromboembolism events were more frequent, in the standard care group (relative risk: 5, 95% CI: 1.1–21.9). 68

Since then, few nomograms, mainly adapted from Raschke's one and using anti-Xa therapeutic ranges, have been tested. 47 82 83 84 85 They confirmed that a weight-based UFH dosing nomogram using anti-Xa monitoring resulted in a high percentage of patients achieving target range 47 62 82 83 85 and that patients monitored with anti-Xa achieved a significantly faster time to therapeutic range and required fewer dose adjustments per 24-hour period, compared with those monitored with aPTT. 47 62 63 However, these nomograms require validation. In such nomograms, a bolus at the initiation of UFH treatment, aimed at saturating the binding of UFH to non-AT proteins (interactome), followed by UFH infusion, and dose adjustments with additional boluses, allowed for effective anticoagulation as fast as possible, despite the complex PK of UFH.

The issue of increased bleeding risk with high UFH doses has been raised by some authors in a cardiopulmonary bypass setting but not really documented, 86 87 88 89 especially the risk of rebound that was assessed using a reagent containing DS, which dissociates protamine–UFH complexes (see above). 89

Therefore, when therapeutic level is not reached or if UFH doses are deemed to be excessively large, before considering a potential action, such as “supplementing“ with AT or resorting to a direct thrombin inhibitor, a careful examination of the case is required. The systematic analysis when facing reduced responsiveness of lab testing to UFH should include coordinated actions between physicians, nurses, and specialists in laboratory medicine in order to: (1) ensure minimization of PF4 release from platelets into collected blood; (2) check whether the drug is appropriately delivered (errors in the preparation, inappropriate infusion rate, defect in the vascular access, concomitant drugs administered through the same line); (3) be aware of analytical issues; (4) be aware of patient characteristics (age, inflammation state); (5) ensure appropriate use of UFH: UFH dose adjustment through a weight-based nomogram (bolus during initiation—dose adjustments, with additional boluses if necessary—timely laboratory monitoring according to nomogram; and (6) consider the possibility of heparin-induced thrombocytopenia, especially in case of associated clinical failure to UFH.

## Scarce Data are Available on Unfractionated Heparin Use beyond Acute Venous Thromboembolism (Key Point #5)

We have highlighted the lack of robust data regarding the prescription and monitoring of UFH during the acute phase of VTE. Beyond VTE, therapeutic doses of UFH are used for managing arterial thrombotic complications (acute coronary syndromes [ACS], perioperative management of acute limb ischemia) and also for preventing thromboses associated with circulatory support (ECMO, left ventricular assistance device) and mechanical heart valves. The doses and monitoring are derived from what is held suitable for acute VTE. Unfortunately, the level of evidence for the use of therapeutic UFH in those clinical situations is extremely low.

Regarding ACS, clinical studies relied on target aPTT values without sound evidence and the desired heparin levels were left unaddressed. In the 2012 ACCP issue, aligned on ACC/AHA guidelines, lower UFH doses than those for VTE are offered for treatment of ACS but without any specified therapeutic range. 8 90

In the management of ECMO, which is used in highly complex critical situations, the aim of anticoagulation is to prevent clotting in the circuit and oxygenator and thrombosis in the ECMO-treated patient while minimizing bleeding complications. Guidance from the International Society on Thrombosis and Haemostasis on the anticoagulation of ECMO patients has been recently published and mostly highlights the lack of robust data. 91 Few studies ara available on this topic: In the setting of venoarterial ECMO a moderate acquired AT deficiency was found, mainly during the first 72 hours, that did not correlate with heparin responsiveness 92 and AT supplementation did not decrease heparin requirement nor diminish the incidence of bleeding and/or thrombosis in adult patients on venovenous ECMO. 93 In this setting, anti-Xa assay compared with aPTT monitoring improved the precision of anticoagulation. 94

To the best of our knowledge, no data are available supporting the way to use UFH for patients with mechanical heart valves, which is managed based on clinical experience.

## Next Steps for Research

We have identified a list of critical points on the use of UFH and laboratory monitoring of therapeutic doses. To improve management of patients receiving UFH, the following issues should be appropriately addressed in future clinical research: (1) while anti-Xa assays are often considered as the preferred option, a vigorous action to improve understanding of the differences between types of anti-Xa assays and to solve the issue of the usefulness of added dextran is necessary; (2) therapeutic ranges for UFH, which were defined decades ago using reagents no longer available, have not been properly validated and need to be confirmed or reestablished; (3) UFH dose adjustment nomograms require full validation.

## Conclusion

In conclusion, most key questions addressed in this review are far from being resolved. Management of patients treated with therapeutic UFH relies on scarce data with questionable clinical relevance, which likely contributes to wide heterogeneity in practice, the more in other settings than acute VTE. Currently used assays for UFH laboratory monitoring display important limitations such as wide variability in the sensitivity of aPTT reagents to UFH and poor harmonization of anti-Xa assays, with questions concerning the addition of dextran.

Not withstanding the limitations and uncertainties of anti-Xa assays, we prefer the use of an anti-Xa assay over aPTT. Most currently used anti-Xa assays are performed without addition of exogenous AT and we do not favor the use of anti-Xa assays supplemented with AT. Such an addition would mask a decreased sensitivity to UFH, but it is unclear to what extent AT dependence of the assay (see above) faithfully reflect the role of AT endogenous levels in heparin response. We call for a vigorous action to improve the availability and use of appropriate and concordant anti-Xa assays and to solve the issue of whether or not to add dextran. Under some circumstances, the aPTT might give an insight on the anticoagulant effect beyond the mere interaction of AT, provided aPTT is not prolonged for other reasons in addition to heparin.

There is an urgent need for additional data with currently used reagents (aPTT or anti-Xa tests) and follow-up of clinical outcomes, to confirm or reestablish therapeutic ranges for UFH. Guidance on UFH management including nomograms, tests, and reagents for monitoring is also required.

## References

[1] Barrowcliffe T W History of heparin Handb Exp Pharmacol 2012 207 3 22 10.1007/978-3-642-23056-1_1 22566218

[2] Hemker H C A century of heparin: past, present and future J Thromb Haemost 2016 14 12 2329 2338 27862941 10.1111/jth.13555

[3] Basu D Gallus A Hirsh J Cade J A prospective study of the value of monitoring heparin treatment with the activated partial thromboplastin time N Engl J Med 1972 287 07 324 327 5041701 10.1056/NEJM197208172870703

[4] Lindahl U Li J P Heparin - An old drug with multiple potential targets in Covid-19 therapy J Thromb Haemost 2020 18 09 2422 2424 32426897 10.1111/jth.14898 PMC7276884

[5] Thachil J Clinical differentiation of anticoagulant and non-anticoagulant properties of heparin J Thromb Haemost 2020 18 09 2424 2425 32470198 10.1111/jth.14933 PMC7283800

[6] Hemker H C Al Dieri R Béguin S Heparins: a shift of paradigm Front Med (Lausanne) 2019 6 254 31803745 10.3389/fmed.2019.00254 PMC6872674

[7] Hirsh J Heparin N Engl J Med 1991 324 22 1565 1574 2027360 10.1056/NEJM199105303242206

[8] Garcia D A Baglin T P Weitz J I Samama M M Parenteral anticoagulants: antithrombotic therapy and prevention of thrombosis, 9th ed: American College of Chest Physicians Evidence-Based Clinical Practice Guidelines Chest 2012 141(2, Suppl):e24S e43S 22315264 10.1378/chest.11-2291 PMC3278070

[9] Ori A Wilkinson M C Fernig D G A systems biology approach for the investigation of the heparin/heparan sulfate interactome J Biol Chem 2011 286 22 19892 19904 21454685 10.1074/jbc.M111.228114 PMC3103365

[10] Hogwood J Mulloy B Lever R Gray E Page C P Pharmacology of heparin and related drugs: an update Pharmacol Rev 2023 75 02 328 379 36792365 10.1124/pharmrev.122.000684

[11] Finley A Greenberg C Review article: heparin sensitivity and resistance: management during cardiopulmonary bypass Anesth Analg 2013 116 06 1210 1222 23408671 10.1213/ANE.0b013e31827e4e62

[12] McMichael A BV Ryerson L M Ratano D Fan E Faraoni D Annich G M 2021 ELSO adult and pediatric anticoagulation guidelines ASAIO J 2022 68 03 303 310 35080509 10.1097/MAT.0000000000001652

[13] Cuker A Unfractionated heparin for the treatment of venous thromboembolism: best practices and areas of uncertainty Semin Thromb Hemost 2012 38 06 593 599 22718256 10.1055/s-0032-1319770

[14] Baluwala I Favaloro E J Pasalic L Therapeutic monitoring of unfractionated heparin - trials and tribulations Expert Rev Hematol 2017 10 07 595 605 28632418 10.1080/17474086.2017.1345306

[15] Levy J H Connors J M Heparin resistance - clinical perspectives and management strategies N Engl J Med 2021 385 09 826 832 34437785 10.1056/NEJMra2104091

[16] Smythe M A Priziola J Dobesh P P Wirth D Cuker A Wittkowsky A K Guidance for the practical management of the heparin anticoagulants in the treatment of venous thromboembolism J Thromb Thrombolysis 2016 41 01 165 186 26780745 10.1007/s11239-015-1315-2 PMC4715846

[17] Alban S From heparins to factor Xa inhibitors and beyond Eur J Clin Invest 2005 35 01 12 20 15701143 10.1111/j.0960-135X.2005.01452.x

[18] Thachil J The versatile heparin in COVID-19 J Thromb Haemost 2020 18 05 1020 1022 32239799 10.1111/jth.14821 PMC9906146

[19] Fabris F Fussi F Casonato A Normal and low molecular weight heparins: interaction with human platelets Eur J Clin Invest 1983 13 02 135 139 6223824 10.1111/j.1365-2362.1983.tb00078.x

[20] Salzman E W Rosenberg R D Smith M H Lindon J N Favreau L Effect of heparin and heparin fractions on platelet aggregation J Clin Invest 1980 65 01 64 73 6243142 10.1172/JCI109661 PMC371340

[21] Messmore H L Griffin B Koza M Seghatchian J Fareed J Coyne E Interaction of heparinoids with platelets: comparison with heparin and low molecular weight heparins Semin Thromb Hemost 1991 17 01 57 59 1712512

[22] Contant G Gouault-Heilmann M Martinoli J L Heparin inactivation during blood storage: its prevention by blood collection in citric acid, theophylline, adenosine, dipyridamole-C.T.A.D. mixture Thromb Res 1983 31 02 365 374 6636048 10.1016/0049-3848(83)90337-7

[23] van den Besselaar A M Meeuwisse-Braun J Jansen-Grüter R Bertina R M Monitoring heparin therapy by the activated partial thromboplastin time–the effect of pre-analytical conditions Thromb Haemost 1987 57 02 226 231 3603414

[24] Gremillet M Talon L Lebreton A Sinegre T Monitoring heparin therapy: stability of two different anti-Xa assays using blood samples collected in citrate-containing and CTAD tubes Thromb J 2023 21 01 21 36803983 10.1186/s12959-023-00465-8 PMC9942401

[25] Toulon P Appert-Flory A Fischer F Buvat S Jambou D Mahagne M H Monitoring unfractionated heparin therapy. 4 hour-stability of anti-Xa activity in unspun citrated tubes Thromb Res 2020 186 7 12 31837560 10.1016/j.thromres.2019.10.019

[26] Billoir P Clavier T Guilbert A Is citrate theophylline adenosine dipyridamole (CTAD) better than citrate to survey unfractionated heparin treatment? Has delayed centrifugation a real impact on this survey? J Thromb Thrombolysis 2019 48 02 277 283 31098816 10.1007/s11239-019-01882-1

[27] Lasne D Toussaint-Hacquard M Delassasseigne C Factors influencing anti-Xa assays: a multicenter prospective study in critically ill and non-critically ill patients receiving unfractionated heparin Thromb Haemost 2023 123 12 1105 1115 37321244 10.1055/s-0043-1770096

[28] Kong F S Zhao L Wang L Ensuring sample quality for blood biomarker studies in clinical trials: a multicenter international study for plasma and serum sample preparation Transl Lung Cancer Res 2017 6 06 625 634 29218266 10.21037/tlcr.2017.09.13 PMC5709139

[29] Magnette A Chatelain M Chatelain B Ten Cate H Mullier F Pre-analytical issues in the haemostasis laboratory: guidance for the clinical laboratories Thromb J 2016 14 49 27999475 10.1186/s12959-016-0123-z PMC5154122

[30] Monroe D M Hoffman M Roberts H R Platelets and thrombin generation Arterioscler Thromb Vasc Biol 2002 22 09 1381 1389 12231555 10.1161/01.atv.0000031340.68494.34

[31] Béguin S Lindhout T Hemker H C The effect of trace amounts of tissue factor on thrombin generation in platelet rich plasma, its inhibition by heparin Thromb Haemost 1989 61 01 25 29 2749591

[32] Mansour A Godier A Lecompte T Roullet S Ten considerations about viscoelastometric tests Anaesth Crit Care Pain Med 2024 43 03 101366 38460888 10.1016/j.accpm.2024.101366

[33] Ranucci M Baryshnikova E Sensitivity of viscoelastic tests to platelet function J Clin Med 2020 9 01 189 32284512 10.3390/jcm9010189 PMC7019879

[34] Bareille M Hardy M Douxfils J Viscoelastometric testing to assess hemostasis of COVID-19: a systematic review J Clin Med 2021 10 08 1740 33923851 10.3390/jcm10081740 PMC8072929

[35] Chandler W L Anticoagulation without monitoring Am J Clin Pathol 2013 140 05 606 607 24124137 10.1309/AJCPE8CWKOVG4AGX

[36] Arachchillage D RJ Kamani F Deplano S Banya W Laffan M Should we abandon the APTT for monitoring unfractionated heparin? Thromb Res 2017 157 157 161 28759760 10.1016/j.thromres.2017.07.006

[37] Hollestelle M J van der Meer F JM Meijer P Quality performance for indirect Xa inhibitor monitoring in patients using international external quality data Clin Chem Lab Med 2020 58 11 1921 1930 32441664 10.1515/cclm-2020-0130

[38] Erdem-Eraslan L Hens J JH van Rossum A P Frasa M AM Keuren J FW Inter-individual variability in phospholipid-dependent interference of C-reactive protein on activated partial thromboplastin time Br J Haematol 2018 183 04 681 683 29143304 10.1111/bjh.15013

[39] Devreese K MJ Verfaillie C J De Bisschop F Delanghe J R Interference of C-reactive protein with clotting times Clin Chem Lab Med 2015 53 05 e141 e145 25324454 10.1515/cclm-2014-0906

[40] Eikelboom J W Hirsh J Monitoring unfractionated heparin with the aPTT: time for a fresh look Thromb Haemost 2006 96 05 547 552 17080209

[41] AGEPS Hemostasis Group Gouin-Thibaut I Martin-Toutain I Peynaud-Debayle E Marion S Napol P Alhenc-Gelas M Monitoring unfractionated heparin with APTT: a French collaborative study comparing sensitivity to heparin of 15 APTT reagents Thromb Res 2012 129 05 666 667 22169771 10.1016/j.thromres.2011.11.016

[42] Hirsh J Raschke R Heparin and low-molecular-weight heparin: the Seventh ACCP Conference on Antithrombotic and Thrombolytic Therapy Chest 2004 126(3, Suppl):188S 203S 15383472 10.1378/chest.126.3_suppl.188S

[43] Toulon P Smahi M De Pooter N APTT therapeutic range for monitoring unfractionated heparin therapy. Significant impact of the anti-Xa reagent used for correlation J Thromb Haemost 2021 19 08 2002 2006 33555096 10.1111/jth.15264

[44] Cuker A Ptashkin B Konkle B A Interlaboratory agreement in the monitoring of unfractionated heparin using the anti-factor Xa-correlated activated partial thromboplastin time J Thromb Haemost 2009 7 01 80 86 19017257 10.1111/j.1538-7836.2008.03224.x

[45] Vandiver J W Vondracek T G Antifactor Xa levels versus activated partial thromboplastin time for monitoring unfractionated heparin Pharmacotherapy 2012 32 06 546 558 22531940 10.1002/j.1875-9114.2011.01049.x

[46] Guervil D J Rosenberg A F Winterstein A G Harris N S Johns T E Zumberg M S Activated partial thromboplastin time versus antifactor Xa heparin assay in monitoring unfractionated heparin by continuous intravenous infusion Ann Pharmacother 2011 45(7-8):861 868 21712506 10.1345/aph.1Q161

[47] Whitman-Purves E Coons J C Miller T Performance of anti-factor Xa versus activated partial thromboplastin time for heparin monitoring using multiple nomograms Clin Appl Thromb Hemost 2018 24 02 310 316 29212374 10.1177/1076029617741363 PMC6714688

[48] Smahi M De Pooter N Hollestelle M J Toulon P Monitoring unfractionated heparin therapy: lack of standardization of anti-Xa activity reagents J Thromb Haemost 2020 18 10 2613 2621 32573889 10.1111/jth.14969

[49] Amiral J Amiral C Dunois C Optimization of heparin monitoring with anti-FXa assays and the impact of dextran sulfate for measuring all drug activity Biomedicines 2021 9 06 700 34205548 10.3390/biomedicines9060700 PMC8235539

[50] Lyon S G Lasser E C Stein R Modification of an amidolytic heparin assay to express protein-bound heparin and to correct for the effect of antithrombin III concentration Thromb Haemost 1987 58 03 884 887 2448889

[51] Hardy M Cabo J Deliège A Gouin-Thibault I Lecompte T Mullier F Reassessment of dextran sulfate in anti-Xa assay for UFH laboratory monitoring Res Pract Thromb Haemost 2023Nov 9;7 08 102257 38193053 10.1016/j.rpth.2023.102257 PMC10772882

[52] Mouton C Calderon J Janvier G Vergnes M C Dextran sulfate included in factor Xa assay reagent overestimates heparin activity in patients after heparin reversal by protamine Thromb Res 2003 111(4-5):273 279 14693175 10.1016/j.thromres.2003.09.014

[53] Hammami E Stiel L Palpacuer C Harzallah I Heparin monitoring during extracorporeal membrane oxygenation: the effect of dextran sulfate on anti-Xa assay Res Pract Thromb Haemost 2023 7 07 102196 38077812 10.1016/j.rpth.2023.102196 PMC10704511

[54] Levine M N Hirsh J Gent M A randomized trial comparing activated thromboplastin time with heparin assay in patients with acute venous thromboembolism requiring large daily doses of heparin Arch Intern Med 1994 154 01 49 56 8267489

[55] Depasse F Gilbert M Goret V Rolland N Samama M M Anti-Xa monitoring: inter-assay variability Thromb Haemost 2000 84 06 1122 1123 11154127

[56] Streng A S Delnoij T SR Mulder M MG Monitoring of unfractionated heparin in severe COVID-19: an observational study of patients on CRRT and ECMO TH Open 2020 4 04 e365 e375 33235946 10.1055/s-0040-1719083 PMC7676995

[57] Barrowcliffe T W Le Shirley Y The effect of calcium chloride on anti-Xa activity of heparin and its molecular weight fractions Thromb Haemost 1989 62 03 950 954 2556814

[58] Tollefsen D M Heparin cofactor II Adv Exp Med Biol 1997 425 35 44 9433487 10.1007/978-1-4615-5391-5_4

[59] Derbalah A Duffull S Newall F Moynihan K Al-Sallami H Revisiting the pharmacology of unfractionated heparin Clin Pharmacokinet 2019 58 08 1015 1028 30850987 10.1007/s40262-019-00751-7

[60] Ruggiero M Melli M Parma B Bianchini P Vannucchi S Isolation of endogenous anticoagulant N-sulfated glycosaminoglycans in human plasma from healthy subjects Pathophysiol Haemost Thromb 2002 32 01 44 49 12214163 10.1159/000057288

[61] Triantos C Louvros E Kalafateli M Endogenous heparinoids detected by anti-Xa activity are present in blood during acute variceal bleeding in cirrhosis. A prospective study J Gastrointestin Liver Dis 2014 23 02 187 194 24949611 10.15403/jgld.2014.1121.232.cht1

[62] Rosborough T K Monitoring unfractionated heparin therapy with antifactor Xa activity results in fewer monitoring tests and dosage changes than monitoring with the activated partial thromboplastin time Pharmacotherapy 1999 19 06 760 766 10391423 10.1592/phco.19.9.760.31547

[63] Zhu E Yuriditsky E Raco V Anti-factor Xa as the preferred assay to monitor heparin for the treatment of pulmonary embolism Int J Lab Hematol 2024 46 02 354 361 37989523 10.1111/ijlh.14207

[64] Benchekroun S Eychenne B Mericq O Heparin half-life and sensitivity in normal subjects and in patients affected by deep vein thrombosis Eur J Clin Invest 1986 16 06 536 539 3104055 10.1111/j.1365-2362.1986.tb02174.x

[65] Chiu H M Hirsh J Yung W L Regoeczi E Gent M Relationship between the anticoagulant and antithrombotic effects of heparin in experimental venous thrombosis Blood 1977 49 02 171 184 831872

[66] Brill-Edwards P Ginsberg J S Johnston M Hirsh J Establishing a therapeutic range for heparin therapy Ann Intern Med 1993 119 02 104 109 8512158 10.7326/0003-4819-119-2-199307150-00002

[67] Wheeler A P Jaquiss R D Newman J H Physician practices in the treatment of pulmonary embolism and deep venous thrombosis Arch Intern Med 1988 148 06 1321 1325 3377615

[68] Raschke R A Reilly B M Guidry J R Fontana J R Srinivas S The weight-based heparin dosing nomogram compared with a “standard care” nomogram. A randomized controlled trial Ann Intern Med 1993 119 09 874 881 8214998 10.7326/0003-4819-119-9-199311010-00002

[69] Hull R D Raskob G E Hirsh J Continuous intravenous heparin compared with intermittent subcutaneous heparin in the initial treatment of proximal-vein thrombosis N Engl J Med 1986 315 18 1109 1114 3531862 10.1056/NEJM198610303151801

[70] Fixed-Dose Heparin (FIDO) Investigators Kearon C Ginsberg J S Julian J A Comparison of fixed-dose weight-adjusted unfractionated heparin and low-molecular-weight heparin for acute treatment of venous thromboembolism JAMA 2006 296 08 935 942 16926353 10.1001/jama.296.8.935

[71] Swayngim R Preslaski C Burlew C C Beyer J Comparison of clinical outcomes using activated partial thromboplastin time versus antifactor-Xa for monitoring therapeutic unfractionated heparin: a systematic review and meta-analysis Thromb Res 2021 208 18 25 34678527 10.1016/j.thromres.2021.10.010

[72] Subcommittee on Plasma Coagulation Inhibitors Van Cott E M Orlando C Moore G W Cooper P C Meijer P Marlar R Recommendations for clinical laboratory testing for antithrombin deficiency; communication from the SSC of the ISTH J Thromb Haemost 2020 18 01 17 22 31894660 10.1111/jth.14648

[73] Lehman C M Rettmann J A Wilson L W Markewitz B A Comparative performance of three anti-factor Xa heparin assays in patients in a medical intensive care unit receiving intravenous, unfractionated heparin Am J Clin Pathol 2006 126 03 416 421 16880140 10.1309/8E3U7RXEPXNP27R7

[74] Croles F N Lukens M V Mulder R de Maat M PM Mulder A B Meijer K Monitoring of heparins in antithrombin-deficient patients Thromb Res 2019 175 8 12 30660948 10.1016/j.thromres.2019.01.007

[75] Ranucci M Baryshnikova E Pistuddi V Di Dedda U The rise and fall of antithrombin supplementation in cardiac surgery Anesth Analg 2023 136 06 1043 1051 36853953 10.1213/ANE.0000000000006314

[76] Béguin S Lindhout T Hemker H C The mode of action of heparin in plasma Thromb Haemost 1988 60 03 457 462 3238649

[77] Swan D Carrier M Lisman T Thachil J Heparin - Messias or Verschlimmbesserung? J Thromb Haemost 2021 19 10 2373 2382 34272818 10.1111/jth.15464 PMC9906358

[78] Gouin-Thibault I Mullier F Lecompte T “Defining heparin resistance: communication from the ISTH SSC Subcommittee of Perioperative and Critical Care Thrombosis and Hemostasis”: comment from Gouin-Thibault et al J Thromb Haemost 2024 22 02 572 574 38309815 10.1016/j.jtha.2023.10.030

[79] Chowdhury V Lane D A Mille B Homozygous antithrombin deficiency: report of two new cases (99 Leu to Phe) associated with arterial and venous thrombosis Thromb Haemost 1994 72 02 198 202 7831651

[80] Tamura S Murata-Kawakami M Takagi Y In vitro exploration of latent prothrombin mutants conveying antithrombin resistance Thromb Res 2017 159 33 38 28961453 10.1016/j.thromres.2017.09.020

[81] Lee H N Cook D J Sarabia A Inadequacy of intravenous heparin therapy in the initial management of venous thromboembolism J Gen Intern Med 1995 10 06 342 345 7562125 10.1007/BF02599954

[82] Lardinois B Hardy M Michaux I Monitoring of unfractionated heparin therapy in the intensive care unit using a point-of-care aPTT: a comparative, longitudinal observational study with laboratory-based aPTT and anti-Xa activity measurement J Clin Med 2022 11 05 1338 35268436 10.3390/jcm11051338 PMC8911237

[83] Smith M L Wheeler K E Weight-based heparin protocol using antifactor Xa monitoring Am J Health Syst Pharm 2010 67 05 371 374 20172987 10.2146/ajhp090123

[84] Rosborough T K In unfractionated heparin dosing, the combination of patient age and estimated plasma volume predicts initial antifactor Xa activity better than patient weight alone Pharmacotherapy 1998 18 06 1217 1223 9855319

[85] Jimaja W E Stirnemann J Fontana P Blondon K S Improving safety of unfractionated heparin: a retrospective, quasi-experimental, observational study of the impact of a pocket card and a computerised prescription aid tool in the University Hospitals of Geneva BMJ Open 2022 12 03 e056912 10.1136/bmjopen-2021-056912 PMC8928257 35292499

[86] Chen Y Phoon P HY Hwang N C Heparin resistance during cardiopulmonary bypass in adult cardiac surgery J Cardiothorac Vasc Anesth 2022 36 11 4150 4160 35927191 10.1053/j.jvca.2022.06.021 PMC9225936

[87] Levy J H Sniecinski R M Reply to the letter to the editor regarding “Defining heparin resistance: communication from the ISTH SSC Subcommittee of Perioperative and Critical Care Thrombosis and Hemostasis” J Thromb Haemost 2024 22 02 575 576 38309816 10.1016/j.jtha.2023.11.004

[88] Levy J H Montes F Szlam F Hillyer C D The in vitro effects of antithrombin III on the activated coagulation time in patients on heparin therapy Anesth Analg 2000 90 05 1076 1079 10781455 10.1097/00000539-200005000-00013

[89] Galeone A Rotunno C Guida P Monitoring incomplete heparin reversal and heparin rebound after cardiac surgery J Cardiothorac Vasc Anesth 2013 27 05 853 858 23627997 10.1053/j.jvca.2012.10.020

[90] Menon V Berkowitz S D Antman E M Fuchs R M Hochman J S New heparin dosing recommendations for patients with acute coronary syndromes Am J Med 2001 110 08 641 650 11382373 10.1016/s0002-9343(01)00715-x

[91] Helms J Frere C Thiele T Anticoagulation in adult patients supported with extracorporeal membrane oxygenation: guidance from the Scientific and Standardization Committees on Perioperative and Critical Care Haemostasis and Thrombosis of the International Society on Thrombosis and Haemostasis J Thromb Haemost 2023 21 02 373 396 36700496 10.1016/j.jtha.2022.11.014

[92] Mansour A Berahou M Odot J Antithrombin levels and heparin responsiveness during venoarterial extracorporeal membrane oxygenation: a prospective single-center cohort study Anesthesiology 2024 140 06 1153 1164 38271619 10.1097/ALN.0000000000004920 PMC11097948

[93] Panigada M Cucino A Spinelli E A randomized controlled trial of antithrombin supplementation during extracorporeal membrane oxygenation Crit Care Med 2020 48 11 1636 1644 32947474 10.1097/CCM.0000000000004590

[94] Hla T TW Christou S Sanderson B Anti-Xa assay monitoring improves the precision of anticoagulation in venovenous extracorporeal membrane oxygenation ASAIO J 2023 70 04 313 320 38039550 10.1097/MAT.0000000000002100

